# Massage and Medical Electricity

**Published:** 1887-09-24

**Authors:** T. S. Dowse


### THE EDITOR'S LETTER-BOX.

### MASSAGE AND MEDICAL ELECTRICITY.

### A CORRECTION.

WILL you in your next issue correct an error which you have inadvertently made in your answer to Nurse Lucas, when you state that Dr. Stretch Dowse lectures on Massage at a "school" connected with the West-end Hospital, 73, Welbeck Street? Allow me to say that I have ceased to be in any way connected with this school of Massage. T. S. DOWSE.

